# Elevated particle number concentrations induce immediate changes in heart rate variability: a panel study in individuals with impaired glucose metabolism or diabetes

**DOI:** 10.1186/s12989-015-0083-7

**Published:** 2015-03-30

**Authors:** Annette Peters, Regina Hampel, Josef Cyrys, Susanne Breitner, Uta Geruschkat, Ute Kraus, Wojciech Zareba, Alexandra Schneider

**Affiliations:** Helmholtz Zentrum München – German Research Center for Environmental Health, Institute of Epidemiology II, Ingolstädter Landstr. 1, 87564 Neuherberg, Germany; German Center for Diabetes Research (DZD e.V.), Neuherberg, Germany; University of Augsburg, Environmental Science Center, Augsburg, Germany; Cardiology Division, University of Rochester Medical Center, Rochester, NY USA

**Keywords:** Epidemiological study, Heart rate variability, Personal exposure, Type 2 diabetes, Ultrafine particles

## Abstract

**Background:**

The health effects of short-term exposure to ambient ultrafine particles in micro-environments are still under investigation.

**Methods:**

Sixty-four individuals with type 2 diabetes and impaired glucose tolerance recorded ambulatory electrocardiograms over five to six hours on 191 occasions in a panel study in Augsburg, Germany. Personal exposure to particle number concentrations (PNC) was monitored for each individual on 5-minute basis concurrently and particulate matter with an aerodynamic diameter < 2.5 μm (PM_2.5_) was acquired from a central monitoring site on an hourly basis.

**Results:**

More than 11,000 5-minute intervals were available for heart rate and measures of heart rate variability including SDNN (standard deviation of NN intervals). A concurrent decrease in 5-minute SDNN of −0.56% (95% confidence limits (CI): −1.02%; −0.09%) and a 5-minute delayed increase in heart rate of 0.23 % (95% CI: 0.11%; 0.36%) was observed with an increase in personal PNC of 16,000 per cm^3^ in additive mixed models. Models evaluating the association of concurrent 5-minute personal PNC and of 1-hour PM_2.5_ showed independent effects on SDNN.

**Conclusion:**

The data suggest that freshly emitted ultrafine particles and aged fine particulate matter are both associated with changes in cardiac function in individuals with type 2 diabetes and impaired glucose tolerance in urban areas.

## Background

Over the past decade, ambient particulate matter has been established as a likely causal risk factor for cardiovascular disease morbidity and mortality [1]. In particular, exacerbation of cardiovascular disease has been observed within individuals with diabetes during episodes of high ambient air pollution exposures [2-4]. It has been noted that ambient particles [5,6] as well as exposure to traffic [7,8] might trigger myocardial infarctions within one or two hours. It is hypothesized that these associations may be a consequence of a direct effect on the electric system of the heart [1]. The effects of air pollution on heart rate (HR) and heart rate variability (HRV) were extensively studied [1] since Pope et al. [9,10], Peters et al. [11], and Gold et al. [12] initially reported these associations. The most consistent evidence with respect to cardiovascular disease exists for fine particulate matter with an aerodynamic diameter smaller than 2.5 μm (PM_2.5_) [1]. Especially, particles from mobile sources are suggested to be linked strongly to cardiovascular disease exacerbation [13]. Particles from emitted mobile sources are much smaller, mostly in the ultrafine range below 100 nm and have the potential to act systemically in organisms [14,15].

Recent evidence from controlled exposures to ultrafine carbon particles suggested altered autonomic function during the exposure in subjects with type 2 diabetes [16]. The study presented here aimed to assess the immediate impact of personal exposure particle to number concentrations (PNC) on HR and HRV measured by ambulatory electrocardiograms (ECG) during five to six hour periods in individuals with diagnosed type 2 diabetes or impaired glucose tolerance (IGT). Specifically, we assess the impact of personally measured PNC during the morning hours on heart rate variability. We build on previous analyses that assessed the association between centrally monitored ambient air pollution and cardiac function within the same study [17]. We had previously reported associations between 1-hour PM_2.5_ and decreased heart rate variability upon concurrent exposure as well as exposures occurring up to 4 hours before the ECG recording.

## Results and discussion

### Patient characteristics

Sixty-four non-smoking panel members were recruited for repeated measurements of personal exposure to PNC and parallel ECG recording. Table 1 describes the baseline characteristics of the 32 individuals with confirmed diagnosis of type 2 diabetes and 32 individuals with IGT recruited based on the KORA F4 study [18,19]. No differences were observed between the type 2 diabetes patients and the individuals with IGT concerning their age, gender, body mass index or disease history. Glycosylated hemoglobin A1c (HbA1c) concentrations above 6.5% were more frequently observed in individuals with diabetes than those with IGT. Diabetes prescriptions were taken by more than half of the participants with diabetes and one participant with IGT. More than 14,000 repeated 5-minute ECG measures and more than 1,200 1-hour ECG measures were available (Table 1). Patients with diabetes had lower HR and HRV on a 5-minute basis. This different was no longer apparent for HRV based on 1-hour ECG recordings.

**Table 1:** **Description of study participants and 5-minute ECG measures**

			**All (N = 64)**		**Type 2 diabetes (N = 32)**			**IGT (N = 32)**			**p-value**
			**Mean**	**(SD)**		**Mean**	**(SD)**		**Mean**	**(SD)**	
Age (years)			66.0	(8.1)		66.8	(6.7)		65.3	(9.4)	0.47^a^
BMI (kg/m^2^)			30.0	(4.7)		30.8	(4.3)		29.3	(4.9)	0.18^a^
			**N**	**(%)**		**N**	**(%)**		**N**	**(%)**	
BMI (kg/m^2^)	≤30		34	(53)		15	(47)		19	(59)	0.32^b^
	>30		30	(47)		17	(53)		13	(41)	
Smoking	never smoker		26	(41)		10	(31)		16	(50)	0.20^c^
	ex smoker		37	(58)		21	(66)		16	(50)	
	occasional smoker		1	(2)		1	(3)		0	(0)	
Employed	no		50	(78)		27	(84)		23	(72)	0.23^b^
	yes		14	(22)		5	(16)		9	(28)	
HbA1c	<6.5%		49	(77)		18	(56)		31	(97)	<0.0001^c^
	≥6.5%		15	(23)		14	(44)		1	(3)	
History of	Coronary heart disease		4	(6)		3	(9)		1	(3)	0.61^c^
	Angina pectoris		5	(8)		2	(6)		3	(9)	1.00^c^
	Myocardial infarction		6	(9)		5	(16)		1	(3)	0.20^c^
	Hypertension		41	(64)		21	(66)		20	(63)	0.79^b^
	None of these diseases		21	(33)		9	(28)		12	(38)	0.42^b^
Medication use	Antidiabetics		18	(28)		17	(53)		1	(3)	<0.0001^c^
	Beta blockers		19	(30)		12	(38)		7	(22)	0.17^b^
	Statins		13	(20)		10	(31)		3	(9)	0.06^c^
	None of the above		28	(44)		6	(19)		22	(69)	<0.0001^c^
ECG		**N**	**Mean**	**(SD)**	**N**	**Mean**	**(SD)**	**N**	**Mean**	**(SD)**	
5 min-intervals	HR	14,874	79.3	(15.6)	7,312	77.7	(15.6)	7,562	80.8	(15.4)	<0.0001^a^
	RMSSD	14,874	30.8	(30.1)	7,312	28.0	(27.3)	7,562	33.5	(32.3)	<0.0001^a^
	SDNN	14,874	47.1	(25.1)	7,312	45.9	(24.6)	7,562	48.2	(25.5)	<0.0001^a^
1 h-intervals	HR	1,203	79.1	(14.4)	597	77.3	(14.4)	606	80.8	(14.1)	<0.0001^a^
	RMSSD	1,203	33.9	(31.7)	597	32.5	(32.2)	606	35.1	(31.2)	0.16^a^
	SDNN	1,203	76.7	(27.1)	597	77.5	(26.0)	606	76.0	(28.2)	0.32^a^

IGT: impared glucose tolerance, SD: standard deviation, BMI: body mass index, HR: heart rate, RMSSD: root mean square of successive differences, SDNN: standard deviation of NN-intervals, ^a^t-test, ^b^Chi-square test, ^c^Fisher’s exact test.

Antidiabetic medications include: insulins and analogues for injection and inhalation.

### Personal exposures to particle number concentrations

**Figure 1:**
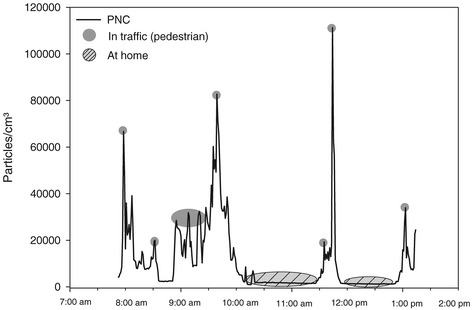
**Example of personal measurements of PNC.** Data was collected starting and ending at the KORA Study Center on November 27^th^ 2007.

Table 2 describes the distribution of the personal PNC measurements and the distribution of particle concentrations at the central monitoring site. Substantially higher variation in personal PNC was observed during personal monitoring compared to the background level (Table 2). Figure 1 describes an example indicating that elevated levels of PNC may occur during times spent in traffic, while indoor concentrations may be substantially lower in the absence of indoor sources. Elevated personal PNC were observed when individuals spent time in traffic (median = 17,884 cm^−3^, N = 3,523), when cooking (median = 43,612 cm^−3^, N = 285) or exposed to environmental tobacco smoke (ETS) (median = 21,929 cm^−3^, N = 148). In contrast, personal PNC concentrations were lower during times spent at home without cooking or ETS exposure (median = 8,833 cm^−3^, N = 6,930). By design of the study, participants were commuting within the urban area of Augsburg in the morning and midday hours. Thereby, personal exposures were impacted by the morning rush-hour as well as by lower traffic volumes during midday and were there deviating from concentrations measured at an urban background monitoring site within the city center. Subject-specific Spearman correlation coefficients between 1-hour personal PNC concentrations and 1-hour ambient ultrafine particles (UFP) had a median of 0.35 and ranged from −0.60 at the 10^th^ percentile to 0.90 at the 90^th^ percentile. Personally measured PNC characterise the exposure to mobile source emissions or other sources of freshly emitted particles and are determined by the personal activities as well as meteorological influences in the region of Augsburg, Germany [20,21].

**Table 2:** **Description of personal 5-minute particle measurements from 191 study visits and 1 hour of ambient particle measurements and meteorology recorded between March 2007 and December 2008**

	**N**	**Mean ± SD**	**Min**	**25%**	**Median**	**75%**	**Max**	**IQR**
**Personal measurements of PNC (5-minute averages)**								
PNC [N/cm^3^]	11,872	20,822 ± 39,233	521	6,354	11,134	21,987	698,225	15,633
**Ambient measurements at stationary monitoring site (1-hour averages)**								
UFP [N/cm^3^]	14,699	9,518 ± 6,902	937	4,892	7,629	12,049	80,858	7,157
ACP [N/cm^3^]	14,699	2,060 ± 1,535	88	1,020	1,657	2,615	17,377	1,595
PM_10_ [μg/m^3^]	15,466	18.3 ± 14.1	0.0	8.4	15.3	24.4	159.8	16.0
PM_2.5_ [μg/m^3^]	15,461	13.7 ± 11.2	0.0	5.8	10.9	18.1	106.5	12.3
Air temperature [°C]	15,398	10.8 ± 7.9	−8.4	4.7	10.8	16.5	33.8	11.8
Relative humidity [%]	15,398	76.9 ± 18.3	21.0	63.3	81.3	92.8	100.0	29.5

SD: standard deviation, IQR: interquartile range, PNC: Particle number concentrations, PM_10_: particulate matter with an aerodynamic diameter <10 μm, PM_2.5_: particulate matter with an aerodynamic diameter <2.5 μm, ACP: accumulation mode particles (100–800 nm), UFP: ultrafine particles (10-100 nm).

Ambient UFP were only moderately correlated with PM_10_ and PM_2.5_ measured at the same central monitoring site (spearman correlation coefficients of 0.49 and 0.42, respectively). In contrast, accumulation mode particles (ACP) were highly correlated to 1-hour PM_10_, PM_2.5_ and UFP (Spearman correlation coefficients of 0.79, 0.75 and 0.70, respectively).

### Changes in heart rate variability in response to particle exposure

Table 3 shows the associations between 5-minute personal exposures to PNC and HR and HRV assessing concurrent and exposures lagged up to 15 minutes. It shows a slightly delayed response of HR and an immediate decrease in SDNN. Different responses of HR and SDNN to PNC may be reasonable given the fact that correlation between HR and SDNN differed substantially between individuals with a median Spearman correlation of −0.10 and a range between −0.53 and 0.55.

**Table 3:** **Associations between personal measurements of 5-minute average particle number concentrations and 5-minute ECG-measures**

	**concurrent**		**0 - 4 min**		**5 - 9 min**		**10 - 14 min**	
	**%-change**	**95% CI**	**%-change**	**95% CI**	**%-change**	**95% CI**	**%-change**	**95% CI**
**HR**	−0.06	−0.18; 0.07	0.23^**^	0.11; 0.36	0.16^*^	0.04; 0.28	−0.01	−0.13; 0.11
**SDNN**	−0.56^*^	−1.02; −0.09	0.36	−0.11; 0.83	0.02	−0.45; 0.48	−0.15	−0.62; 0.32
**RMSSD**	−0.13	−0.74; 0.48	0.08	−0.54; 0.70	0.14	−0.48; 0.77	−0.16	−0.77; 0.46

Analyses considered concurrent and up to 15-minutes delayed exposures and adjusted for trend, meteorology and time of day. Effect estimates are shown for an increase of 16,000 particles cm^−3^.

*p-value <0.05, **p-value <0.01, CI: confidence interval, HR: heart rate, RMSSD: root mean square of successive differences, SDNN: standard deviation of NN intervals.

**Figure 2:**
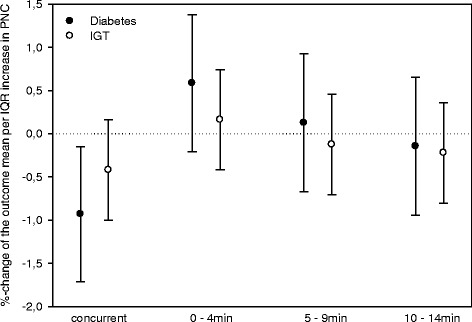
**Effects of personally measured 5-minute PNC on SDNN based on 5-minute ECG recordings in patients with diabetes or impaired glucose tolerance.** Effect estimates are shown for an increase of 16,000 particles cm^−3^.

Associations between PNC and SDNN appear to be more pronounced in individuals with diabetes than in individuals with IGT (Figure 2). Exploratory analyses extending the time-lag between 5-minute personal exposure to PNC and HR, SDNN or RMSSD up to one hour showed no consistent pattern beyond 15 minutes.

We had previously shown associations between 1-hour ambient air pollution concentrations and cardiac function occurring up to a lag of 4 hours [17]. We had chosen one hour intervals of exposure and ECG recordings *a priori* as we considered this the minimal time scale for a central monitoring site in an urban background location to represent population average exposures. In Table 4 we compare the association between 1-hour averages of personal PNC and ambient UFP, ACP, PM_10_ and PM_2.5_ and concurrent measures of HR and HRV over 1-hour. No consistent associations between personal or ambient particles number concentrations (PNC, UFP, ACP) and HR were observed. In contrast, PM_10_ and PM_2.5_ were associated both with SDNN and RMSSD as reported previously [17]. The association between PM_2.5_ and HRV was stronger in individuals with IGT than those with type 2 diabetes, but the differences did not achieve statistical significance. In line with our results, Chan and colleagues observed significant decreases in SDNN and RMSSD in association with an increase of 10,000 particles/cm^3^ in personally measured particles in the size range between 20 nm and 1 μm in a prospective panel study [22]. Adverse changes in HR and HRV were also observed in association with ambient UFP in panel or cross-over studies [23-28] and with concentrated UFP in controlled chamber studies [29,30] albeit some associations were not significant. However, some studies reported no or even positive associations between HRV and UFP [31-33].

**Table 4:** **Associations between ambient 1**-**hour average air pollution concentrations at the central monitoring site and 1**-**hour average ECG-measures**

	**HR**		**SDNN**		**RMSSD**	
	**%-change**	**95% CI**	**%-change**	**95% CI**	**%-change**	**95% CI**
Personal PNC	0.13	−0.19; 0.45	−0.93^†^	−2.01; 0.16	0.53	−0.70;1.77
UFP	0.40	−0.16; 0.95	0.99	−0.66; 2.64	−0.12	−2.40; 2.21
ACP	0.35	−0.39; 1.09	−0.30	−2.23; 1.64	−1.58	−5.19; 2.18
PM_10_	0.67	−0.20; 1.54	−2.78^*^	−4.98; −0.59	−5.00^*^	−8.88; −0.95
PM_2.5_	0.63	−0.44; 1.71	−3.27^*^	−5.84; −0.69	−6.86^**^	−11.73; −1.72

Analyses considered concurrent exposures and adjusted for trend, meteorology and time of day. Effect estimates are shown for an increase in interquartile range as given in Table 2.

^†^p-value <0.1, *p-value <0.05, **p-value <0.01, CI: confidence interval, HR: heart rate, RMSSD: root mean square of successive differences, SDNN: standard deviation of NN-intervals, PNC: Particle number concentrations, PM_10_: particulate matter with an aerodynamic diameter <10μm, PM_2.5_: particulate matter with an aerodynamic diameter <2.5μm, UFP: ultrafine particles (10-100μm); ACP: accumulation mode particles (100-800 nm).

**Figure 3:**
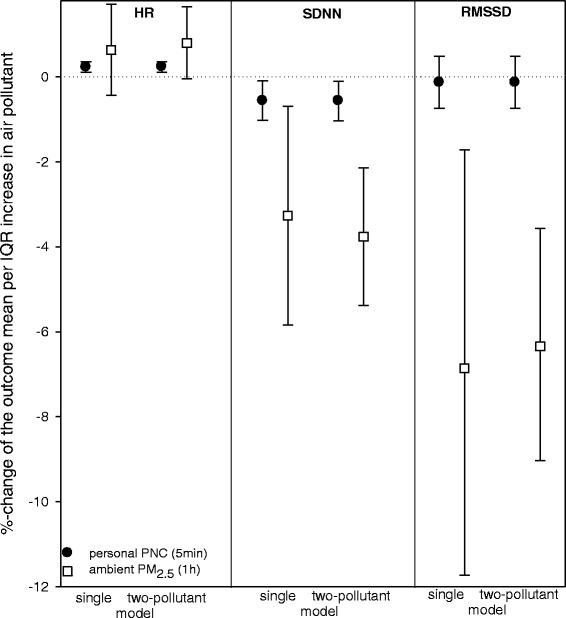
**Two pollutant models for 5-minute personal PNC and 1-hour ambient PM**_**2.5**_ **on 5-minute HR and HRV parameters. in patients with diabetes or impaired glucose tolerance.** Effect estimates are shown for an increase of 16,000 particles cm^−3^ and 12 μg m^−3^ PM_2.5_.

Effect estimates were larger for the 1-hour PM_2.5_ than for personal PNC and associations between 1-hour PM_2.5_ concentrations and 5-minute HRV strengthened when adjusting for personal PNC (Figure 3). PM_2.5_ measured at an urban background monitoring site quantifies the overall particulate matter level predominantly determined by the meteorological conditions. In the present study, we demonstrate therefore that particle exposures determined by personal proximity to sources and by urban background levels both are associated with changes in cardiac function on a very immediate time scale.

Earlier studies have observed associations between hourly concentrations of PM_2.5_ and the onset of myocardial infarction in Boston, MA [5] and Rochester, NY [6]. Moreover, times spent in traffic were associated with the onset of myocardial infarction [7,8] and controlled exposure studies suggest that effects of diesel exposures might be enhanced by exercise [34]. Previous studies have in many instances indicated that personal exposures to PM_2.5_ or to gaseous pollutants are associated with changes in HRV [26,35-51]. The study participants ranged from healthy adults to patients with cardiovascular diseases or asthma and were studied in different settings around the world. We had chosen individuals with impaired glucose metabolism because individuals with type 2 diabetes had been shown to be susceptible to air pollution [2-4]. A study of controlled human exposures to concentrated ultrafine particles showed immediate effects on subjects with metabolic syndrome, however, did not observe changes in HRV one hour after the exposure [30]. In contrast, in a study in subjects with type 2 diabetes indicated a decrease in the high frequency component of heart rate variability and increased heart rates persisting up to 48 hours [16]. Furthermore, there is an emerging body of evidence linking ambient air quality as one of the risk factors to type 2 diabetes [52]. Data from controlled animal experiments [53] as well as analyses in prospective population-based cohort studies [54-58] support this association. Systemic inflammation, activation of innate immunity in the lung and an imbalance of the autonomic nervous system induced by air pollution exposures jointly potentially provide the link to insulin resistance and diabetes exacerbation [52]. Sudden changes in cardiac function may predispose susceptible individuals to sudden cardiac deaths during episodes with elevated particle concentrations [59]. Most likely, different underlying intrinsic mechanisms are activated by 5-minute PNC and 1-hour PM_2.5_. We hypothesize that shortly elevated PNC may activate irritant receptors and lead thereby to changes in the autonomic control [60]. In contrast, we hypothesize that the changes in HRV observed in association with PM_2.5_ are associated with an activation of host defense on an alveolar level, which may involve translocation of particle components, immediate systemic oxidative stress response and an activation of leukocytes [52].

### Sensitivity analyses

**Figure 4:**
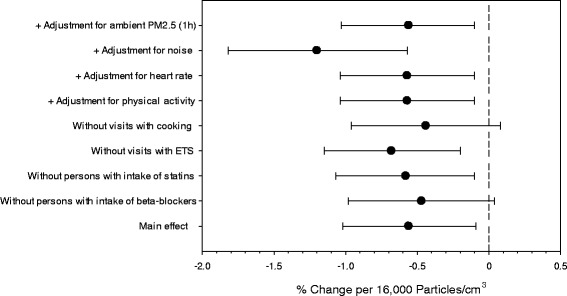
**Sensitivity analyses of the association between concurrent exposure to personally measured PNC and SDNN.** *Regression coefficient as reported in Table 3.

Associations were robust in sensitivity analyses and a summary is given in Figure 4 for the association between personally measured personal PNC and SDNN. No statistically significant difference was observed in individuals without beta-blockers intake or statin use. By selecting individuals with impaired glucose tolerance, we intended to study the impact of particles in individuals who were not heavily treated by beta-blockers or statins as these medications may obliterate the effects of particle exposures [61,62].

Excluding time periods when the participants recorded ETS exposures or cooking rendered consistent results, but suggested that indoor sources contributed to the observed associations. We employed two different ways to adjust for physical activity. Neither adjusting for the diary entries of physical activity nor for heart rate did change the effect estimates. Models including personal noise exposure showed stronger associations with personal PNC (Figure 3) and increased 5-minute SDNN (3.35% [95% CI: 2.95% ; 4.11%] per 5 db[A]) as reported previously [63]. These analyses suggested that the associations of PNC and noise with ECG-parameters were potentially confounding each other. To further test the impact of the model choices, we conducted sensitivity analyses for the immediate effect of PNC on SDNN. Including a time trend within the measurements or including the previous segments of SDNN as a predictor did not change the effect estimates substantially (5-minute SDNN: −0.56% [−0.98%;-0.13%] or −0.42% [−0.77%;-0.06%] per 16,000 cm^−3^ PNC, respectively).

### Limitations

The study assessed personal measurements of PNC which is a novel marker for personal exposure to fresh combustion particles. The study thereby overcomes one large limitation of previous panel studies. By employing direct measurements of PNC it also provides different and novel information compared to studies of personal PM_2.5_ or gaseous pollutants [26,35-49]. However, the measurement devices are usually operated by technical personnel to measure indoor and outdoor particle concentrations and were not designed for study participants. As a consequence we were only able to achieve 80% of the planned hourly measurements albeit stringent examiner training, review of the instruction sessions by audiotape, and written instructions for the participants. The missing measurements had no certain pattern and were related to diligence in following the instructions by the study participants. Diaries were kept by the participants, but no geographic positioning system data was acquired. ECG data and personal PNC data were processed independently. While the examiners and the participants were aware of the study hypotheses, information on their HR was not available and levels of PNC were not discussed with respect to limit or guideline values as these do not exist.

Timing of the measurements were based on recorded times from the instruments and the study protocols. Discrepant times were checked individually, discussed with the study nurses and corrected wherever possible.

Each day’s measurement provided control data for the individual and correlation within the day and the individual was considered. Analyses proved to be relatively robust against other assumptions of the covariance structure. Confounding by physical activity, a potentially important individual time-varying factor was considered but did not prove to be strong and resulted in changes of the effect estimates of less than 10%.

There were no statistically significant differences with respect to age, body mass index, HbA1c concentrations, history of cardiovascular disease and medication use when comparing the study participants to all individuals with either diabetes or IGT in the underlying sample of the KORA cohort study. Participants of the panel study were more likely to be unemployed, many of them already retired. In addition, the proportion of ex-smokers was higher in the present study than in the overall sample.

As this study is assessing short-term impacts of urban area ambient particulate matter, it does not address the question, whether long-term exposure to particulate matter is associated with an increased risk for incident diabetes as recently shown [54-58]. However, the data reported here provides evidence that short-term exposure to ambient particulate matter may contribute to cardiovascular disease exacerbation in individuals with impaired glucose metabolism or diabetes.

## Conclusion

The data presented here shows changes in HRV associated with personally measured PNC and ambient PM_2.5_ suggesting that both freshly emitted ultrafine particles as well as aged aerosol in urban areas are associated with changes in cardiac function. The study suggests that personal activities and elevated particle concentrations in micro-environments may modify personal exposures and thereby impact on cardiac function. The study was conducted in individuals with type 2 diabetes and IGT suggesting that these subgroups of the population might be at risk for cardiovascular disease exacerbation when transiently exposed to fresh and aged urban particulate matter.

## Methods

### Study design

A prospective panel study was conducted in Augsburg, Germany, between March 19, 2007 and December 17, 2008. Individuals with diabetes mellitus type 2 or impaired glucose tolerance (IGT) were recruited from an ongoing examination of 3,080 individuals as part of the KORA F4 cohort study (Cooperative Health Research in the Region of Augsburg) as described in detail elsewhere [18,19]. Type 2 diabetes was defined on based on a validated physician diagnosis, or newly diagnosed diabetes (≥7.0 mmol⁄l fasting or ≥ 11.1 mmol⁄l 2-h glucose) determined by an oral glucose tolerance test. IGT was defined according to the 1999 World Health Organization diagnostic criteria [64]. Exclusion criteria for the present study were 1) current active smoking, 2) intake of platelet aggregation inhibitors except for acetylsalicylic acid, 3) a myocardial infarction and/or interventional procedure (PTCA, bypass surgery) less than 6 months before the beginning of the study, and 4) chronic inflammatory diseases such as Crohn’s disease, colitis ulcerosa, and rheumatoid arthritis. Furthermore, individuals were not included in case of 1) an implanted pacemaker, 2) atrial fibrillation, 3) allergy to latex, and 4) thrombosis or shunt in an arm to standardize HRV analyses. All individuals participated in repeated visits scheduled every 4–6 weeks on the same weekday and at the same time of the day.

### Ethics and consent statement

The study was conducted in compliance with the Helsinki Declaration. Ethical approval for the study was granted by the Ethics Committee of the Bayerische Landesärztekammer, München, Germany. The study protocol including the participant information and the consent form were part of the ethics review. The study participants gave informed written consent before entering into the study.

### ECG monitoring

In the personal monitoring program, participants were equipped for five to six hours with an electrocardiogram (ECG) device during their second up to the fifth visit as described previously [17]. ECGs were recorded with a 12-lead Mortara H12 digital Holter recorder (Mortara Instrument, Milwaukee, WI, USA). Analyses of heart rate variability were restricted to ECGs that had at least 200 beats available for 5-minute intervals. Heart rate (HR) and time domain parameters of HRV, the standard deviation of all normal-to-normal (NN) intervals (SDNN), and the root mean square of successive NN interval differences (RMSSD) were determined on a 5-minute and an hourly basis. Only individuals with at least one ECG recording with duration of at least two hours were used for analysis.

### Personal particle number concentration monitoring

Personal exposure to PNC was measured using a portable condensation particle counter model 3007 (TSI Inc., USA) which covered a diameter range from 10 nm to 1 μm. Participants were instructed on how to restart the measurements if tilting might have resulted in an automated stop of the measurements. They carried the device in a specially designed carrier bag within an inlet at the top. Moreover, participants were asked to keep a diary on their activities during the 5–6 hours of personal measurements including information on times spent indoors or outdoors, times spent in traffic, indoor activities such as cooking and sources such as environmental tobacco smoke exposures (ETS). The participants were instructed to always keep the device close by, but at least within the same room at a central location. Diary information was checked for plausibility and used to process the measurement data. In four instances, participants did not carry the PNC device with them for short periods of time (8 minutes, 10 minutes, 30 minutes or 1 hour). These data were excluded from the analyses. Usually, measurements started around 7:30 a.m.; participants were free to go wherever they liked and returned at around 1 p.m. Three portable condensation particle counters were employed during the study. All of them were serviced before the start of the study and comparison measurements were conducted in March 2007. Additional service periods were conducted every six months. More detail is provided in [20].

### Central site air pollution monitoring

Ambient air pollution was measured at a central measurement site in Augsburg throughout the complete study period as described previously [65,66]. The measurement location was in urban background approximately 1 km to the south-east of the city center. Particle mass concentrations of PM_2.5_ and PM_10_ (particulate matter ≤ 2.5 or 10 μm in aerodynamic diameter, respectively) were measured by two separate Tapered Element Oscillating Microbalance (TEOM, model 1400ab, Thermo Fisher Scientific Inc., USA). To correct the PM measurements for aerosol volatility effects, each TEOM was equipped with a Filter Dynamics Measurement System (FDMS, model 8500b, Thermo Fisher Scientific Inc., USA). Particle size distributions in the range from 3–900 nm were measured by a custom-built Twin Differential Mobility Particle Sizer (TDMPS) system consisting of two cylindrical, Vienna-type Differential Mobility Analyzers (DMA) covering complementary size ranges (3 to 23 nm as well as 18 to 900 nm). For the analysis we used the size fraction of ultrafine particles from 10 to 100 nm (ambient UFP) and of accumulation mode particles from 100 to 800 (ambient ACP).

### Statistical analyses

Repeated continuous outcome data was analyzed using mixed models with random patient effects to accommodate repeated measures and to account for unobserved heterogeneity of the data. To account for dependencies of the outcome measures, covariance structure considered autocorrelation of the first order for measurements of the same day and correlation between measurements of the same individual at days apart. This was done within the framework of additive mixed models to allow for semi-parametric and non-parametric exposure-response functions. Models were selected separately for HR, SDNN, and RMSSD as described previously [17]. Final models included for HR: time trend (linear), time of day (morning vs. afternoon), 1-hour air temperature (lag 2, polynomial of degree 2), 1-hour relative humidity (lag 1, linear); for SDNN: time trend (linear), time of day (morning vs. afternoon), 1-hour air temperature (concurrent, linear), 1-hour relative humidity (concurrent, linear) ; and for RMSSD: time trend (linear), time of day (morning vs. afternoon), 1-hour air temperature (lag 7, linear), 1-hour relative humidity (lag 4, linear).

Models were adjusted for ambient meteorology and temporal trends. Penalized splines were used to allow for non-linear confounder adjustment. Results are presented as %-change from the mean per 16,000 ultrafine particles cm^−3^ or the respective interquartile ranges together with 95% confidence intervals. A number of sensitivity analyses were conducted including models adjusting for personal 5-minute noise exposure measured as A-weighted equivalent continuous sound pressure levels (*L*eq) reported in units of A-weighted decibels [dB(A)] (Spark® model 703; Larson Davis Inc., Depew, NY, USA) as described elsewhere [63]. Data were analyzed using SAS statistical software (version 9.1; SAS Institute Inc., Cary, NC, USA).
